# Proteome-wide epitope mapping identifies a resource of antibodies for SARS-CoV-2 detection and neutralization

**DOI:** 10.1038/s41392-021-00573-9

**Published:** 2021-04-24

**Authors:** Te Liang, Mengli Cheng, Fei Teng, Hongye Wang, Yongqiang Deng, Jiahui Zhang, Chengfeng Qin, Shubin Guo, Hui Zhao, Xiaobo Yu

**Affiliations:** 1 State Key Laboratory of Proteomics, Beijing Proteome Research Center, National Center for Protein Sciences-Beijing (PHOENIX Center), Beijing Institute of Lifeomics, Beijing, 102206 China; 2 Department of Virology, State Key Laboratory of Pathogen and Biosecurity, Beijing Institute of Microbiology and Epidemiology, Academy of Military Medical Sciences, Beijing, 100071 China; 3 Emergency Medicine Clinical Research Center, Beijing Chao-Yang Hospital, Capital Medical University, & Beijing Key Laboratory of Cardiopulmonary Cerebral Resuscitation, Beijing, 100020 China

**Keywords:** Infectious diseases, Infection

**Dear Editor,**

Since December 2019, the coronavirus disease 2019 (COVID-19) caused by the severe acute respiratory syndrome coronavirus-2 (SARS-CoV-2) has become a worldwide pandemic.^1^ Significant efforts have been made to generate antibodies to help study COVID-19 pathogenesis, perform diagnostic testing, and develop treatment to neutralize SARS-CoV-2 activity. However, the specific sequence of amino acids recognized and bound by an antibody, the “epitope” is unknown for most antibodies. In this study, we developed a high-throughput epitope mapping platform using a peptide-based SARS-CoV-2 proteome microarray.^2^ to detect the binding epitopes of 57 commercial antibodies to ORF1ab, nucleocapsid (N), spike (S), envelop (E), membrane (M), ORF3a, ORF6, ORF7a, and ORF8 proteins (Supplementary Table S1).

The workflow of antibody epitope mapping and applications are shown in Fig. 1a. Our peptide-based proteome microarray contained 966 peptides representing all SARS-CoV-2 proteins. Each peptide was 15 amino acids long with a 5 amino acid overlap. The intra- and inter-array correlation of IgG antibody detection were 0.9960 and 0.9945, respectively (Supplementary Fig. S1), demonstrating a high degree of reproducibility of the SARS-CoV-2 proteome microarray. Epitope mapping of 57 commercial antibodies was performed as previously described.^2^ All microarray data were normalized to a *Z*-score and immune-reactive peptides were selected using *Z*-score > 1.96 (95% confidence interval).

**Fig. 1:**
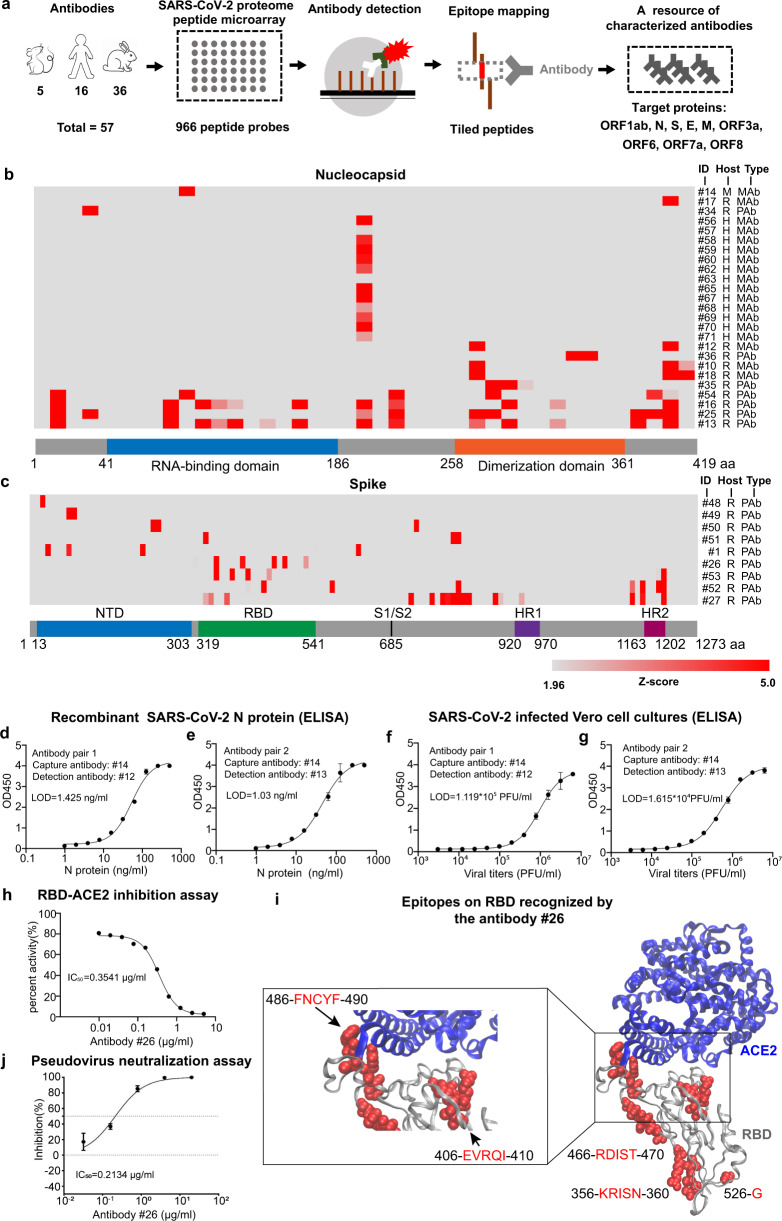
The epitope profiling database constructed by using a peptide-based SARS-CoV-2 proteome microarray and its application. **a** Schematic illustration of epitope mapping using a peptide-based SARS-CoV-2 proteome microarray. **b**, **c** Distribution of antibody epitopes within the N and S proteins respectively. The *x*-axis represents the protein sequence. The *y*-axis represents the identification (ID) number (#number), host (mouse = M, rabbit = R, human = H) and clonality of the antibody (polyclonal antibody = PAb, monoclonal antibody = MAb). **d**, **e** Detection of the purified, full-length recombinant N protein using ELISA prepared with two antibody pairs selected from epitope mapping analyses. **f**, **g** Detection of the N protein from inactivated SARS-CoV-2 in cell culture using ELISA prepared with two antibody pairs selected from epitope mapping analyses. **h** The neutralization activity of antibody #26 using an S-RBD-ACE2 inhibition assay. **i** Structural analysis of the antibody #26 epitopes on SARS-CoV-2 RBD (PDB ID: 6m0j). **j** Neutralization activities of antibody #26 using a pseudovirus assay

The binding epitopes of the antibodies analyzed in this study are shown in Supplementary Table S2. The linear epitopes of 18 monoclonal antibodies and 7 polyclonal antibodies targeted sequences within the N protein. Sixteen of these antibodies (#13, #16 #25 #56-#60, #62, #63, #65, and #67–#71) have the same epitope (206-SPARM-210) (Fig. 1b). However, a mouse monoclonal antibody (#14) targets 96-GGDGK-100 (Fig. 1b). In addition, nine antibodies have multiple binding epitopes, five of which (#12, #36, #10, #18, and #35) recognize the C-terminal domain while the epitopes of four antibodies (#54, #16, #25, and #13) are distributed along with the entire N protein (Fig. 1b). One rabbit polyclonal antibody (#34) has only one epitope: 36-RSKQR-40 (Fig. 1b).

For the S protein, 53% (9/17) of the antibodies bound to peptides on our array (Fig. 1c). The other 47% (8/17) that did not bind to any peptide may have low affinity or target conformational or post translational modifications (PTMs),^3^ which are not present on our array that is comprised of chemically-synthesized peptides. Antibodies #26 and #53 targeted regions within the receptor-binding domain (RBD) of S protein (Fig. 1c). Antibody #53 also targeted an epitope on the C-terminus. The epitopes of antibodies #48 and #49 are located within the N-terminal domain (Fig. 1c).

The epitopes of 15 antibodies to the SARS-CoV-2 ORF1ab, M, E, ORF6, ORF7a, and ORF8 proteins were also analyzed (Supplementary Table S2 and Fig. S2). Our analyses indicate that the epitopes of ORF1ab antibodies are located on nsp1, nsp2, nsp3, nsp12, and nsp13 proteins (Supplementary Fig. S2a). The epitopes of two ORF3a antibodies (#45 and #46) are 266-EPTTTTSVPL-275, 176-TSPIS-180, respectively. Another ORF3a antibody (#47) has two epitopes, including 246-IHTID-250 and 266-EPTTTTSVPL-275 (Supplementary Fig. S2b). Two rabbit polyclonal anti-M antibodies (#42, #43) bind to 186-RVAGDSGFAAYSRYR-200 and 206-LNTDH-210, respectively (Supplementary Fig. S2c). The E protein antibody (#44) has two epitopes, including 16-SVLLF-20 and 56-FYVYSRVKNLNSSRVPDLLV-75 (Supplementary Fig. S2d). The epitope (46-ENKYSQLDEEQPMEID-61) of ORF6 protein antibody (#41) is located at the C-terminus (Supplementary Fig. S2e). Two rabbit polyclonal antibodies (#39, #40) to the ORF7a protein bind to 46-FHPLA-50 and 86-LFIRQEEVQELYSPI-100, respectively (Supplementary Fig. S2f). Two rabbit polyclonal antibodies (#37 and #38) to the ORF8 protein target the 66-GSKSP-70 and 106-EDFLE-110 epitopes, respectively (Supplementary Fig. S2g).

As a structural protein, the N protein is an ideal target for SARS-CoV and SARS-CoV-2 detection due to its high expression and immunogenicity. Currently, nucleic acid and serum antibody testing have remained the major techniques for COVID-19 diagnostics. Previously, the detection of the N protein has been found as a sensitive serum biomarker (90% positive) in early SARS infection, which was superior to nucleic acid (42.9%) and serum antibodies (21.4%).^4^ To demonstrate the utility of epitope profiling for COVID-19 diagnosis, we identified a monoclonal antibody (#14) with high specificity to the epitope (96-GGDGK-100) on the N protein (Supplementary Fig. S3a). As such, we used this antibody as a capture antibody for a sandwich-based enzyme-linked immunosorbent assay (ELISA) due to the high specificity. In addition, we identified two antibodies (#12 and #13) containing multi epitopes (Supplementary Fig. S3b, c), which are not overlapped with that of antibody #14. Thus, we speculate that antibodies #12 and #13 are good to be used to the detection antibody in ELISA. To test our hypothesis, we tested the capture antibody #14 with two detection antibodies (#12, #13), representing two antibody pairs (pair 1: #14 and #12, pair 2: #14 and #13). Using serial dilutions of purified, full-length recombinant N protein, the ELISA had a detection range from 1 to 500 ng/ml. The limit of detection (LOD) was 1.425 and 1.03 ng/ml for antibody pairs 1 and 2, respectively (Fig. 1d, e). The average intra-variations were 3.57% and 9.83%, whereas the average inter-variations were 12.55% and 11.35% for antibody pairs 1 and 2, respectively. To determine whether our ELISAs could detect the native N protein, we infected Vero cells with SARS-CoV-2 virus for 2–3 days and then used our ELISA to detect the N protein in cell culture inactivated at 56 °C for 60 min. The native SARS-CoV-2 N protein was detected using our ELISAs with a dynamic range of ~3 orders of magnitude, with a LOD of 1.119 × 10^5^ PFU/ml (pair 1) and 1.615 × 10^4^ PFU/ml (pair 2) (Fig. 1f, g). These results indicate the great potential of our ELISAs in detecting SARS-CoV-2 in the clinic.

The SARS-CoV-2 S protein, via its RBD, mediates viral binding to the angiotensin-converting enzyme 2 (ACE2) and entry into host cells.^1^ As such, much effort has been made to develop therapeutic antibodies or vaccines that target the S protein or the RBD domain. We analyzed the neutralization activity of two anti-S protein antibodies (#26 and #53) with linear epitopes on the RBD using a SARS-CoV-2 spike RBD-ACE2 interaction inhibitor assay. A rabbit polyclonal antibody (#26) showed significant inhibition activity with an IC_50_ of 0.3541 µg/ml (Fig. 1h). Five epitopes of #26 antibody (356-KRISN-360, 406-EVRQI-410, 466-RDIST-470, 486-FNCYF-490, and 526-GPKKS-530) were within the RBD domain (Fig. 1i), which included six amino acids (E406, Q409, F486, N487, Y489, and F490) previously shown to be involved in SARS-CoV-2 neutralization.^1,5^ To validate this hypothesis, we tested the neutralization activity of #26 antibody using a SARS-CoV-2 pseudovirus system. Interestingly, the #26 antibody displayed strong inhibition activity to pseudovirus infection with an IC_50_ of 0.2134 µg/ml (Fig. 1j). We also randomly tested two Mouse/Human chimeric anti-S-RBD antibodies (#20, #21) that did not bind to any S peptides on the array. The IC_50_ of #20 and #21 were 0.8846 and 4.016 µg/ml, respectively (Supplementary Fig. S4a, b), which are much higher than that of #26 antibody. As mentioned above, these antibodies may bind to conformational epitopes or PTMs.^3^

Altogether, our results show that our peptide-based SARS-CoV-2 proteome microarray can characterize the epitope landscape of SARS-CoV-2 antibodies. Identification of SARS-CoV-2 antigenic sequences may help reveal the different B-cell responses to SARS-CoV-2 in different species and develop reagents for COVID-19 diagnosis and treatment.

## Supplementary information

SUPPLEMENTAL MATERIAL

## Data Availability

All data can be available upon the request of the corresponding author.
