# Supplementary material for: Proteome-wide epitope mapping identifies a resource of antibodies for SARS-CoV-2 detection and neutralization
Source: Signal Transduct Target Ther. 2021 Apr 24;6:166. doi: 10.1038/s41392-021-00573-9 (PMC8065321; doi:10.1038/s41392-021-00573-9)
Supplement: Supplementary file 1 — SUPPLEMENTAL MATERIAL [file 41392_2021_573_MOESM1_ESM.pdf]

-Supplementary Information-

Proteome-wide epitope mapping identifies a resource of antibodies for SARS-CoV-2 detection and neutralization

Te Liang[1], Mengli Cheng[2], Fei Teng[3], Hongye Wang[1], Yongqiang Deng[2], Jiahui Zhang[1], Chengfeng Qin[2], Shubin Guo[3*], Hui Zhao[2*], Xiaobo Yu[1*]

[1] State Key Laboratory of Proteomics, Beijing Proteome Research Center, National Center for Protein Sciences-Beijing (PHOENIX Center), Beijing Institute of Lifeomics, Beijing, 102206, China.

[2] Department of Virology, State Key Laboratory of Pathogen and Biosecurity, Beijing Institute of Microbiology and Epidemiology, Academy of Military Medical Sciences, Beijing 100071, China

[3] Emergency Medicine Clinical Research Center, Beijing Chao-Yang Hospital, Capital Medical University, & Beijing Key Laboratory of Cardiopulmonary Cerebral Resuscitation, Beijing 100020, China.

[*]Correspondence: yuxiaobo@mail.ncpsb.org (X. Y.), shuishu2002@126.com (H. Z.), shubinguo@126.com (S. G.)

This file includes:

Materials and Methods

Fig. S1 to S4

Table S1 and S2

Materials and methods

Characterization of anti-SARS-CoV / SARS-CoV-2 antibodies using a SARS-CoV-2 proteome microarray

The SARS-CoV-2 proteome microarray containing full-length N, full-length E, truncated S proteins, and 966 peptides representing all SARS-CoV-2 proteins was prepared as described previously[1]. The array was assembled in an incubation tray and blocked with 5% (w/v) milk in phosphate-buffered saline (PBS) containing 0.05% (v/v) Tween-20 (PBST) for 30 min at room temperature before antibody detection. After aspirating the blocking solution, the commercial antibody (0.5 μg/ml) was added to the array and incubated at room temperature for 20 min. After washing three times with PBST, the array was then incubated for 20 min with a Cy™3 Affinipure donkey anti-human IgG (H+L) antibody (Jackson ImmunoResearch, USA) (2 μg/ml), or an Alexa Fluor 555 labeled goat anti-rabbit IgG (H+L) antibody (Invitrogen, USA) (2 μg/mL), or an Alexa Fluor 555 Goat anti-mouse IgG (H+L) antibody (Invitrogen, USA) (0.5 μg/mL). Finally, the array was washed with PBST and deionized water, dried with vacuum pump and disassembled from the tray. The proteome microarray was scanned at 532 nm using a GenePix 4300A microarray scanner (Molecular Devices). The median fluorescent signal intensity of each spot with background subtraction was extracted using GenePix Pro7 software (Molecular Devices).

Detection of SARS-CoV-2 nucleocapsid protein using ELISA

96-well half area clear flat bottom polystyrene high-binding microplates (Corning, USA) were coated with the antibody #14 (Sino Biological, Inc., China, Cat: 40143-MM05) (1 μg/ml) at 4 °C overnight and blocked in 0.2% PBST with 5% skim milk for 1 h at 37 °C after washing. The diluted recombinant SARS-CoV-2 N protein (Sino Biological, Inc., China, Cat: 40588-V08B) or the inactivated SARS-CoV-2 IME-BJ01 strain[2] in cell culture was added to the wells, and the plates were incubated at 37 °C for 1 h. After the wells were washed, antibody #12 (Sino Biological, Inc., China, Cat: 40143-R001) or #13 (Sino Biological, Inc., China, Cat: 40143-RP01) was added at a 1:1000 dilution. After 1 h at 37 °C, the wells were washed. A horseradish peroxidase (HRP) conjugated goat anti-rabbit IgG antibody (CoWin Biosciences, China) was added to the wells, and the plates were incubated at 37 °C for 1 h. After washing with PBST, 3', 3', 5', 5', -tetramethylbenzidine (TMB) (CoWin Biosciences, China) was added to the wells, and the reaction was stopped by adding ELISA Stop Solution (Solarbio, China). After briefly shaking the plates, the absorbance at 450 nm was read.

The SARS-CoV-2-ACE2 inhibition assay

The SARS-CoV-2 spike RBD-ACE2 interaction inhibitor assay was conducted as described previously with modification[3]. 96-well microplates (Corning, USA) were coated with the SARS-CoV-2 spike RBD recombinant protein (Sino Biological, Inc., China, Cat: 40592-V05H) (1 μg/ml) at 4°C

overnight and blocked in 0.2% PBST with 5% skim milk for 1 h at room temperature after washing. The serially diluted antibodies were added to the wells， and the plates were incubated at room temperature for 1 h. After the wells were washed, the recombinant human ACE2 protein with a poly-histidine tag at the C-terminus (Sino Biological, Inc., China, Cat: 10108-H08H) was added, incubated for 1 h at room temperature, and washed again. HRP conjugated anti His-tag monoclonal antibody (CoWin Biosciences, China) was added and the plates were incubated at room temperature for 1 h. After washing with PBST, TMB (CoWin Biosciences, China) was added to the wells, and the reaction was stopped by adding ELISA Stop Solution (Solarbio, China). After briefly shaking the plates, the absorbance at 450 nm was read.

Pseudovirus-based neutralization assay.

The pseudovirus assay was conducted as described previously[2, 4]. Huh7 cells were seeded in 96-well plates at a concentration of $2*10^4$ cells per well, then incubated until 90-100% confluency for approximately 24 h. The pseudovirus expressing the SARS-CoV-2 S protein was incubated with serial 5-fold dilutions of antibody for 1 h at 37 °C, and dulbecco's modified eagle medium (DMEM) was used as a negative control. The mixture was added to cultured cells for infection and incubated for 24 h. The cell supernatant was removed and luciferase was activity measured using a GloMax 96® Microplate

Luminometer (Promega). Percent inhibition was calculated relative to the “no antibody” control.

Data analysis

Prior to the data analysis, all microarray signals were normalized using a Z-score, in which a threshold of 1.96 was used as the criteria for identifying peptide epitopes. Antibody response to the peptides was visualized as a heatmap using the MultiExperiment Viewer software[5]. Statistical analyses were performed using the GraphPad Prism software and Microsoft Excel. Immunogenic epitopes on the 3D structure of viral proteins were annotated by using the VMD 1.9.3.

References

1. Wang., H. et al. SARS-CoV-2 proteome microarray for mapping COVID-19 antibody interactions at amino acid resolution. *ACS Cent. Sci.*, 10.1021/acscentsci.1020c00742 (2020).
2. Zhang, N.N. et al. A Thermostable mRNA Vaccine against COVID-19. *Cell* **182**, 1271-1283 e1216 (2020).
3. Esparza, T., Martin, N., Anderson, G., Goldman, E. & Brody, D. High Affinity Nanobodies Block SARS-CoV-2 Spike Receptor Binding Domain Interaction with Human Angiotensin Converting Enzyme. *BioRxiv* (2020).
4. Nie, J. et al. Establishment and validation of a pseudovirus neutralization assay for SARS-CoV-2. *Emerg Microbes Infect* **9**, 680-686 (2020).
5. Chu, V.T., Gottardo, R., Raftery, A.E., Bumgarner, R.E. & Yeung, K.Y. MeV+R: using MeV as a graphical user interface for Bioconductor applications in microarray analysis. *Genome Biol* **9**, R118 (2008).

Supplementary Figures

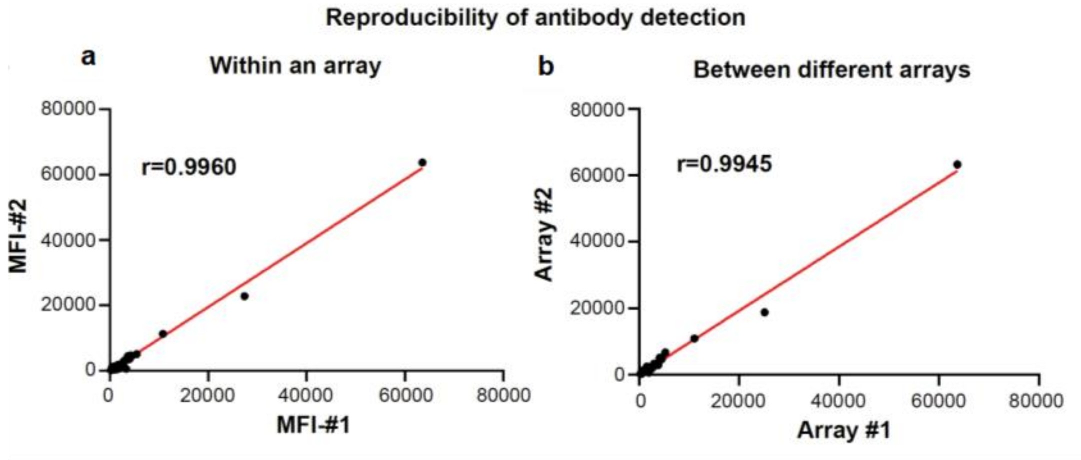

Fig. S1. The reproducibility of antibody detection using the SARS-CoV-2 proteome microarray. (a) and (b) are the reproducibility of antibody detection within an array and across different arrays. The r correlation was calculated within an array and across between different arrays.

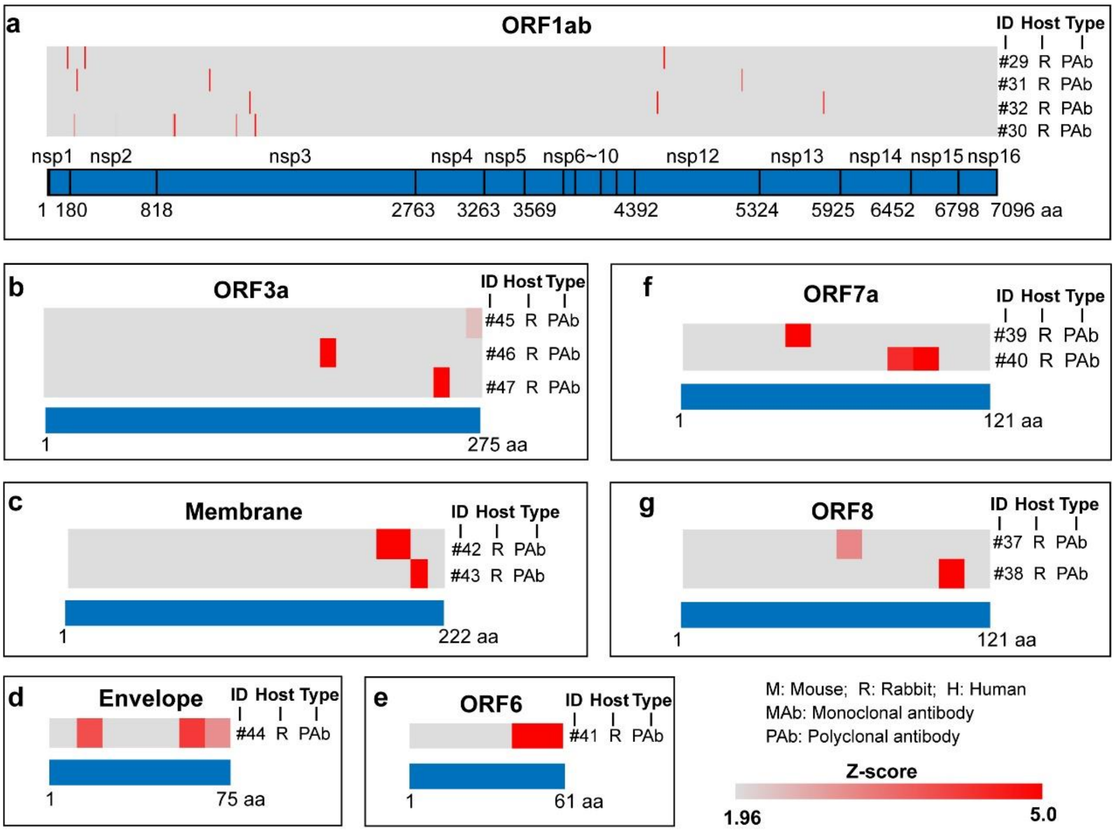

Fig.S2. A landscape of antibody epitopes among SARS-CoV-2 proteins. (a-g) Distribution of antibody epitopes with the ORF1ab, ORF3a, M, E, ORF6, ORF7a, and ORF8 proteins, respectively. The x-axis represents the protein sequence. The y-axis represents the identification (ID) number (#number), host (mouse = M, rabbit = R, human = H) and clonality of the antibody (polyclonal antibody = PAb, monoclonal antibody = MAb).

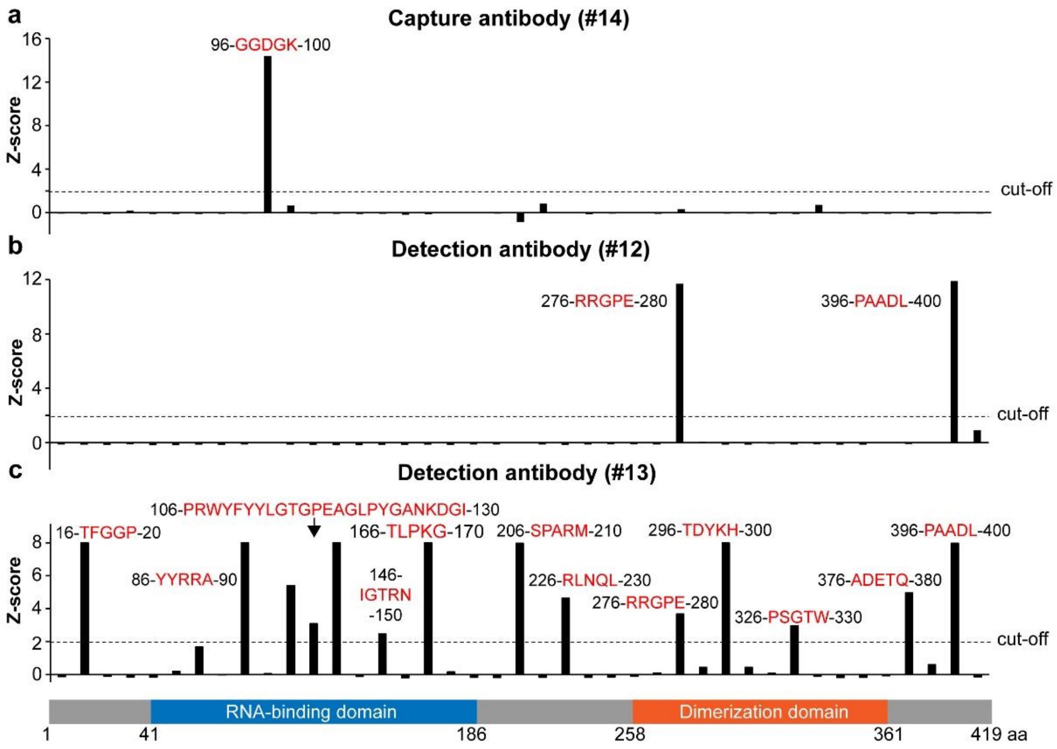

Fig.S3. The epitope profiles of the antibodies selected for the ELISA. (a-c) Epitopes on N protein of the capture antibody #14 and two detection antibodies (#12, #13), respectively.

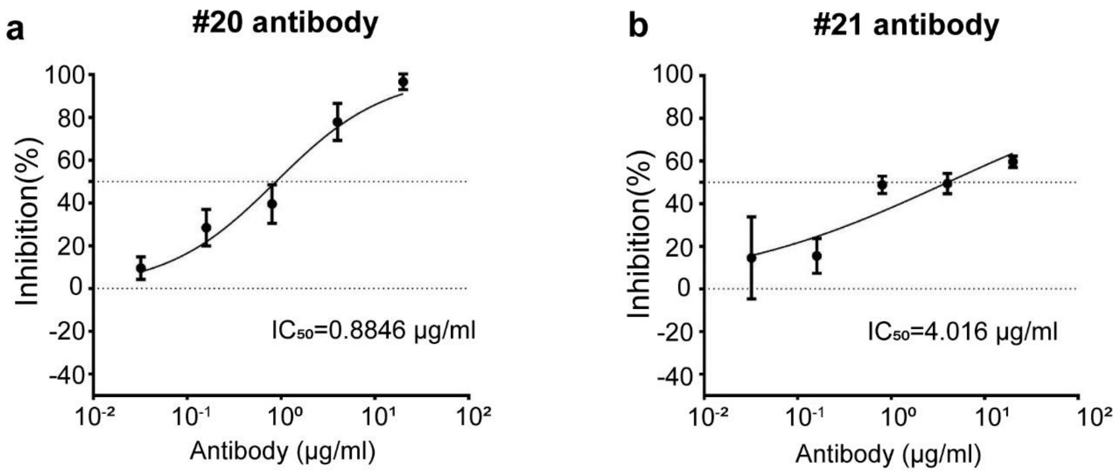

Fig.S4. Characterization of antibody neutralization activity. (a) and (b) are the neutralization activities of antibodies #20 and #21 using a pseudovirus assay, respectively.

Supplementary Tables

Table S1. List of antibodies used for the epitope mapping analyses

ID	Company[a]	Catalog No.	Specificity	MAb/PAb[b]	Host
#1	SB	40150-T62	SARS-CoV S1 subunit	PAb	Rabbit
#9	SB	40150-R007	SARS-CoV-2 S1 subunit	MAb	Rabbit
#10	SB	40143-R019	SARS-CoV-2 N protein	MAb	Rabbit
#12	SB	40143-R001	SARS-CoV N protein	MAb	Rabbit
#13	SB	40143-RP01	SARS-CoV N protein	PAb	Rabbit
#14	SB	40143-MM05	SARS-CoV N protein	MAb	Mouse
#16	SB	40143-T62	SARS-CoV N protein	PAb	Rabbit
#17	SB	40143-R004	SARS-CoV N protein	MAb	Rabbit
#18	SB	40143-R040	SARS-CoV N protein	MAb	Rabbit
#20	SB	40150-D001	SARS-CoV S protein RBD	MAb	Mouse/ Human chimeric
#21	SB	40150-D002	SARS-CoV S protein RBD	MAb	Mouse/ Human chimeric
#22	SB	40150-D003	SARS-CoV S protein RBD	MAb	Mouse/ Human chimeric
#23	SB	40150-D004	SARS-CoV S protein RBD	MAb	Mouse/ Human chimeric
#25	SB	40588-T62	SARS-CoV-2 N protein	PAb	Rabbit
#26	SB	40592-T62	SARS-CoV-2 S protein RBD	PAb	Rabbit
#27	SB	40590-T62	SARS-CoV-2 S2 subunit	PAb	Rabbit
#29	BT	NCP0001	SARS-CoV-2 ORF1ab polyprotein	PAb	Rabbit
#30	BT	NCP0002	SARS-CoV-2 ORF1ab polyprotein	PAb	Rabbit

#31	BT	NCP0003	SARS-CoV-2 ORF1ab polyprotein	PAb	Rabbit
#32	BT	NCP0004	SARS-CoV-2 ORF1ab polyprotein	PAb	Rabbit
#34	BT	NCP0006	SARS-CoV-2 N protein	PAb	Rabbit
#35	BT	NCP0007	SARS-CoV-2 N protein	PAb	Rabbit
#36	BT	NCP0008	SARS-CoV-2 N protein	PAb	Rabbit
#37	BT	NCP0009	SARS-CoV-2 ORF8 protein	PAb	Rabbit
#38	BT	NCP0010	SARS-CoV-2 ORF8 protein	PAb	Rabbit
#39	BT	NCP0011	SARS-CoV-2 ORF7a protein	PAb	Rabbit
#40	BT	NCP0012	SARS-CoV-2 ORF7a protein	PAb	Rabbit
#41	BT	NCP0013	SARS-CoV-2 ORF6 protein	PAb	Rabbit
#42	BT	NCP0014	SARS-CoV-2 M protein	PAb	Rabbit
#43	BT	NCP0015	SARS-CoV-2 M protein	PAb	Rabbit
#44	BT	NCP0016	SARS-CoV-2 E protein	PAb	Rabbit
#45	BT	NCP0017	SARS-CoV-2 ORF3a protein	PAb	Rabbit
#46	BT	NCP0018	SARS-CoV-2 ORF3a protein	PAb	Rabbit
#47	BT	NCP0019	SARS-CoV-2 ORF3a protein	PAb	Rabbit
#48	BT	NCP0020	SARS-CoV-2 S protein	PAb	Rabbit
#49	BT	NCP0021	SARS-CoV-2 S protein	PAb	Rabbit
#50	BT	NCP0022	SARS-CoV-2 S protein	PAb	Rabbit
#51	BT	NCP0023	SARS-CoV-2 S protein	PAb	Rabbit
#52	BT	NCP0032	SARS-CoV-2 S protein ECD	PAb	Rabbit

#53	BT	NCP0033	SARS-CoV-2 S protein RBD	PAb	Rabbit
#54	BT	NCP0034	SARS-CoV-2 N protein	PAb	Rabbit
#56	BT	HMN001	SARS-CoV-2 N protein	MAb	Human
#57	BT	HMN002	SARS-CoV-2 N protein	MAb	Human
#58	BT	HMN003	SARS-CoV-2 N protein	MAb	Human
#59	BT	HMN004	SARS-CoV-2 N protein	MAb	Human
#60	BT	HMN005	SARS-CoV-2 N protein	MAb	Human
#62	BT	HMN007	SARS-CoV-2 N protein	MAb	Human
#63	BT	HMN008	SARS-CoV-2 N protein	MAb	Human
#65	BT	HMN010	SARS-CoV-2 N protein	MAb	Human
#67	BT	HMN012	SARS-CoV-2 N protein	MAb	Human
#68	BT	HMN013	SARS-CoV-2 N protein	MAb	Human
#69	BT	HMN014	SARS-CoV-2 N protein	MAb	Human
#70	BT	HMN015	SARS-CoV-2 N protein	MAb	Human
#71	BT	HMN016	SARS-CoV-2 N protein	MAb	Human
#72	BT	HMB001	SARS-CoV-2 S protein ECD/RBD	MAb	Human
#73	BT	HMB002	SARS-CoV-2 S protein ECD/RBD	MAb	Human
#75	BT	HMB004	SARS-CoV-2 S protein ECD	MAb	Human

[a] SB and BT are abbreviations for Sino Biological, Inc., and Bioworld Technology, Co. respectively

[b] MAb and PAb are abbreviations for monoclonal antibody and polyclonal antibody, respectively

Table S2. Binding epitopes of antibodies identified with the SARS-CoV-2 proteome array

Antibody ID	Target protein	Peptides	Epitopes
#29	ORF1ab	orf1ab-16	156-DFQEN-160
		orf1ab-29	286-PRVEK-290
		orf1ab-461	4606-GNWYD-4610
#31	ORF1ab	orf1ab-23	226-RGVYC-230
		orf1ab-122	1216-TESKP-1220
		orf1ab-519	5186-MSEAK-5190
#32	ORF1ab	orf1ab-152	1516-YSGQS-1520
		orf1ab-456	4556-DFVEN-4560
		orf1ab-580	5796-FKMFY-5800
#30	ORF1ab	orf1ab-21	206-ARAGK-210
		orf1ab-52	516-AKKGA-520
		orf1ab-95	946-QYEYGTEDDYQGKPL-960
		orf1ab-96	
		orf1ab-142	1416-VDYGA-1420
		orf1ab-156	1556-LKTLL-1560
#48	S	S-3	26-PAYTN-30

#49	S	S-8	76-TKRFDNPVLPFNDGV-90
		S-9	
#50	S	S-24	236-TRFQTLLALHRSYLT-250
		S-25	
		S-74	736-VDCTM-740
#51	S	S-34	336-CPFGE-340
		S-81	806-LPDPSKPSKRSFIED-820
		S-82	
#1	S	S-4	36-VYYPD-40
		S-8	76-TKRFD-80
		S-22	216-LPQGF-220
		S-55	546-LTGTG-550
		S-63	626-ADQLT-630
#26	S	S-32	316-SNFRV-320
		S-36	356-KRISN-360
		S-41	406-EVRQI-410
		S-47	466-RDIST-470
		S-49	486-FNCYF-490
		S-53	526-GPKKS-530
#53	S	S-36	356-KRISN-360
		S-39	386-KLNDL-390
		S-42	416-GKIAD-420

		S-46	456-FRKSN-460
		S-120	1196-SLIDLQELGKYEQYI-1210
		S-121	
#52	S	S-42	416-GKIAD-420
		S-45	446-GGNYN-450
		S-58	576-VRDPQ-580
		S-82	816-SFIED-820
		S-115	1146-DSFKE-1150
		S-117	1166-LGDIS-1170
		S-120	1196-SLIDLQELGKYEQYI-1210
		S-121	
#27	S	S-34	336-CPFGEVFNATRFASV-350
		S-35	
		S-38	376-TFKCY-380
		S-73	726-ILPVS-730
		S-77	766-ALTGI-770
		S-79	786-KQIYKTPPIKD
		S-80	FGGFNFSQIL
		S-81	PDPSKPSKR
		S-82	SFIEDLLFNK
		S-83	VTLADAGFI
		S-84	KQYGDC-840

		S-89	886-WTFGAGAALQIPFAM-900
		S-90	
		S-94	936-DSLSS-940
		S-115	1146-DSFKE-1150
		S-117	1166-LGDIS-1170
		S-121	1206-YEQYI-1210
#45	ORF3a	ORF3a-27	266-EPTTTTSVPL-275
#46	ORF3a	ORF3a-18	176-TSPIS-180
#47	ORF3a	ORF3a-25	246-IHTID-250
		ORF3a-27	266-EPTTTTSVPL-275
#44	E	E-2	16-SVLLF-20
		E-6	56-FYVYSRVKNLNSSRVPDLLV-75
		E-7	
#42	M	M-19	186-RVAGDSGFAAYSRYR-200
		M-20	
#43	M	M-21	206-LNTDH-210
#41	ORF6	ORF6-5	46-ENKYSQLDEEQPMEID-61
		ORF6-6	
#39	ORF7a	ORF7a-5	46-FHPLA-50
#40	ORF7a	ORF7a-9	86-LFIRQEEVQELYSPI-100
		ORF7a-10	
#37	ORF8	ORF8-7	66-GSKSP-70

#38	ORF8	ORF8-11	106-EDFLE-110
#14	N	N-10	96-GGDGK-100
#17	N	N-40	396-PAADL-400
#34	N	N-4	36-RSKQR-40
#56	N	N-21	206-SPARM-210
#57	N	N-21	206-SPARM-210
#58	N	N-21	206-SPARM-210
#59	N	N-21	206-SPARM-210
#60	N	N-21	206-SPARM-210
#62	N	N-21	206-SPARM-210
#63	N	N-21	206-SPARM-210
#65	N	N-21	206-SPARM-210
#67	N	N-21	206-SPARM-210
#68	N	N-21	206-SPARM-210
#69	N	N-21	206-SPARM-210
#70	N	N-21	206-SPARM-210
#71	N	N-21	206-SPARM-210
#12	N	N-28	276-RRGPE-280
		N-40	396-PAADL-400
#36	N	N-34<br>N-35	336-AIKLDDKDPNFKDQV-350

#10	N	N-28	276-RRGPE-280
		N-40	396-PAADLDDFSKQLQQSMSSADSTQA-419
		N-41	
#18	N	N-28	276-RRGPE-280
		N-40	396-PAADLDDFSKQLQQSMSSADSTQA-419
		N-41	
#35	N	N-29	286-FGDQELIRQGTDYKHWPQIAQFAPS-310
		N-30	
		N-31	
#54	N	N-2	16-TFGGP-20
		N-10	96-GGDGK-100
		N-23	226-RLNQL-230
		N-29	286-FGDQE-290
		N-39	386-QKKQQTVTLLPAADL-400
		N-40	
#16	N	N-2	16-TFGGP-20
		N-9	86-YYRRA-90
		N-11	106-PRWYFYYLGTGPEAGLPYGANKDGI-130
		N-12	
		N-13	
		N-17	166-TLPKG-170
		N-21	206-SPARM-210

		N-23	226-RLNQL-230
		N-30	296-TDYKH-300
		N-33	326-PSGTW-330
		N-40	396-PAADL-400
#25	N	N-2	16-TFGGP-20
		N-4	36-RSKQR-40
		N-9	86-YYRRA-90
		N-17	166-TLPKG-170
		N-21	206-SPARM-210
		N-23	226-RLNQL-230
		N-28	276-RRGPEQTQGNFGDQE-290
		N-29	
		N-38	376-ADETQALPQRQKKQQTVTLLPAADL-400
		N-39	
		N-40	
#13	N	N-2	16-TFGGP-20
		N-9	86-YYRRA-90
		N-11	106-PRWYFYYLGTGPEAGLPYGANKDGI-130
		N-12	
		N-13	
		N-15	146-IGTRN-150
		N-17	166-TLPKG-170

N-21	206-SPARM-210
N-23	226-RLNQL-230
N-28	276-RRGPE-280
N-30	296-TDYKH-300
N-33	326-PSGTW-330
N-38	376-ADETQ-380
N-40	396-PAADL-400
